# Early Functional Improvement in American Spinal Injury Association Impairment Scale Grade C (AIS C) Non-Traumatic Spinal Cord Injury Following Staged Thoracic Decompression and Inpatient Rehabilitation: A Case Report

**DOI:** 10.7759/cureus.115971

**Published:** 2026-09-08

**Authors:** Ramiz K Ahmed, Sarah Shandy, Harjas Jassal, Tha Cha, Duc Chung

**Affiliations:** 1 Medical School, California Health Sciences University College of Osteopathic Medicine, Clovis, USA; 2 Physical Medicine and Rehabilitation, San Joaquin Valley Rehabilitation Hospital, Fresno, USA; 3 Palliative Care, University of California, San Francisco Fresno, Fresno, USA; 4 Palliative Care, California Health Sciences University, Clovis, USA

**Keywords:** ais c, asia impairment scale, functional recovery, gait recovery, inpatient rehabilitation, multidisciplinary rehabilitation, neuroplasticity, non-traumatic spinal cord injury, spinal cord injury rehabilitation, thoracic spinal stenosis

## Abstract

Non-traumatic spinal cord injury (NTSCI) may result from degenerative spinal conditions, including spinal stenosis and multilevel disc herniation, and can cause substantial motor, sensory, and functional impairment. We present a 33-year-old man with NTSCI secondary to severe thoracic spinal stenosis and multilevel disc herniation. Before the initial surgical intervention, the available neurological examination documented 5/5 upper-extremity strength and 4/5 strength in the bilateral lower extremities. Sensation was impaired below a thoracic level, and hyperreflexia and other findings consistent with thoracic myelopathy were documented. Gait was impaired, while bowel and bladder function remained preserved. A complete segment-by-segment International Standards for Neurological Classification of Spinal Cord Injury (ISNCSCI) examination and formal preoperative ASIA Impairment Scale (AIS) classification were not documented; therefore, a preoperative lower-extremity motor score could not be reliably calculated. During a 26-day inpatient rehabilitation stay, the patient progressed from non-ambulatory status with extensive assistance for mobility and self-care to assisted ambulation of 170 feet with a front-wheeled walker and contact guard assistance, accompanied by improvements in transfers, wheelchair mobility, and activities of daily living. Although objective neurological findings provide important context for the patient’s course, complete serial ISNCSCI motor and sensory scores and standardized rehabilitation outcome measures were unavailable; therefore, the principal documented outcome is functional improvement rather than quantified neurological recovery. The patient’s trajectory was likely multifactorial, reflecting surgical relief of mechanical compression, early postoperative and spontaneous neurological recovery, preserved motor pathways associated with motor-incomplete SCI, psychological engagement, and rehabilitation-facilitated functional adaptation.

## Introduction

Spinal cord injury (SCI) can impair sensory, motor, and autonomic function and may result in substantial long-term disability. Non-traumatic spinal cord injury (NTSCI) is etiologically diverse and commonly results from degenerative spinal disease, spinal stenosis, tumors, infection, or vascular pathology. A 2024 systematic review and meta-analysis estimated a pooled global NTSCI incidence of 17.93 cases per million population while emphasizing substantial geographic and methodological heterogeneity [1]. Registry data further demonstrate that patients with NTSCI differ from those with traumatic SCI in age, comorbidity burden, etiology, and clinical presentation [2].

Neurological level and severity are classified using the International Standards for Neurological Classification of Spinal Cord Injury (ISNCSCI) and the ASIA Impairment Scale (AIS) [3]. AIS C denotes a motor-incomplete injury. Motor-incomplete status requires either voluntary anal contraction or sensory-incomplete status with motor function preserved more than three levels below the motor level on either side. In AIS C, more than half of the key muscle functions below the single neurological level of injury have muscle grades less than 3/5 [3].

Motor-incomplete injuries have greater recovery potential than motor-complete injuries, although prognosis varies according to baseline AIS grade, neurological level, age, lower-extremity motor function, and other clinical factors. In a multicenter cohort of older adults with AIS C SCI, 64.2% improved to AIS D, and 7.1% improved to AIS E [4]. In a population-based cohort of traumatic cervical SCI, initial AIS C-D status and higher lower-extremity motor scores were associated with later ambulation; ambulation included walking with or without an assistive device [5]. A recent systematic review and meta-analysis of traumatic SCI similarly identified baseline neurological severity, neurological level, and motor function as important predictors of walking recovery, although direct extrapolation to NTSCI should be made cautiously [6].

Neurological recovery and functional recovery are related but distinct concepts. Changes in motor and sensory function reflect the neurological state of the spinal cord, whereas functional recovery describes the patient’s ability to translate available neurological capacity into mobility, transfers, self-care, and participation in daily activities. During the early postoperative period, improvement may reflect multiple overlapping processes, including relief of mechanical compression, spontaneous or postoperative neurological recovery, and rehabilitation-facilitated functional adaptation [7-10]. Specialized inpatient rehabilitation provides coordinated medical management, rehabilitation nursing, physical therapy, occupational therapy, caregiver training, and discharge planning during this period [7,8].

This case describes the neurological context and substantial functional progression of a patient with motor-incomplete NTSCI following staged thoracic decompression and stabilization, with particular emphasis on his 26-day inpatient rehabilitation course.

## Case presentation

A 33-year-old man with obesity, hypertension, obstructive sleep apnea, and depression, who had previously been independent in activities of daily living, developed progressive bilateral lower-extremity weakness, impaired ambulation, sensory disturbance, and declining functional independence in the setting of severe thoracic spinal stenosis.

Before the initial surgical intervention, the available neurological examination documented 5/5 upper-extremity strength with bilateral lower-extremity weakness in the approximately 4/5 range. Sensation was impaired below a thoracic level, and hyperreflexia and other findings consistent with thoracic myelopathy were documented. Gait was impaired, while bowel and bladder function remained preserved. A complete segment-by-segment ISNCSCI examination and formal preoperative AIS classification were not documented; therefore, a preoperative lower-extremity motor score could not be reliably calculated.

The patient underwent T1-T2 laminectomy and facetectomy for decompression, posterior fusion, and instrumentation on February 4, 2026. He was subsequently admitted to inpatient rehabilitation on February 9. On February 11, he developed progressive left-greater-than-right lower-extremity weakness with a new left foot drop and worsening gait impairment, ultimately becoming unable to ambulate. Further evaluation identified multilevel thoracic disc herniation with severe spinal cord compression. The patient was transferred back to acute care and subsequently underwent T1-T7 posterior bilateral instrumented fusion and laminectomies on February 14, 2026.

Representative postoperative CT imaging obtained after the initial T1-T2 procedure and before the subsequent T1-T7 operation demonstrated upper thoracic instrumentation (Figure 1). Preoperative imaging and imaging obtained after the final T1-T7 posterior fusion and laminectomies were not available for inclusion.

**Figure 1:**
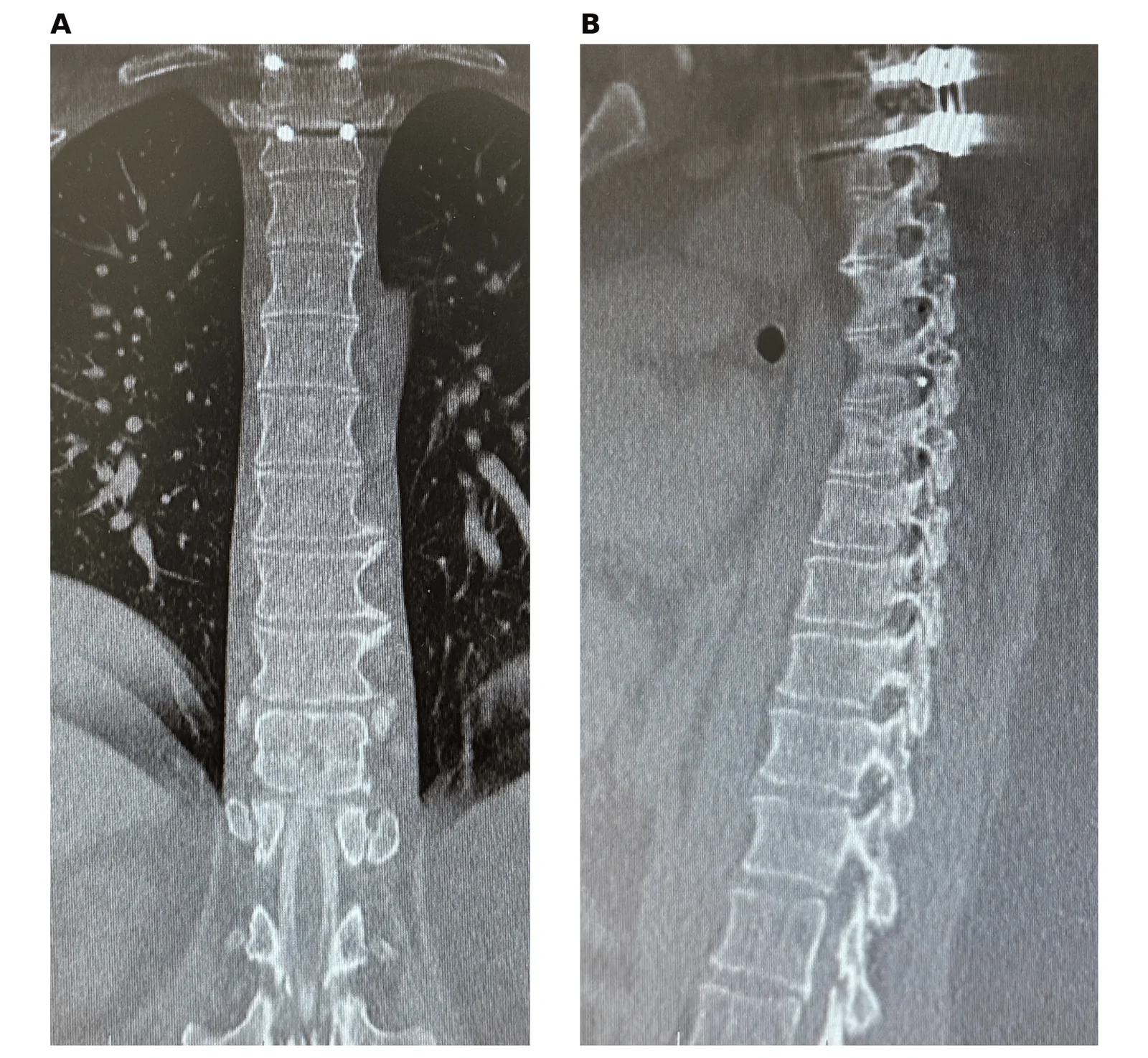
Postoperative CT imaging following the initial T1-T2 decompression and fusion. (A) Coronal CT view demonstrating upper thoracic spinal instrumentation following the initial T1-T2 decompression and fusion. (B) Sagittal CT view demonstrating postoperative upper thoracic alignment and fusion hardware following the initial procedure. Preoperative imaging and imaging following the subsequent T1-T7 posterior fusion and laminectomies were not available for inclusion.

Following medical stabilization, the patient was readmitted to inpatient rehabilitation on February 22, 2026. At that time, the injury was documented as T10 AIS C. Upper-extremity strength was 5/5 throughout. In the right lower extremity, hip flexion was 2/5, knee extension was 3/5, and ankle dorsiflexion was 3/5. In the left lower extremity, hip flexion was 2/5, knee extension was 2/5, and ankle dorsiflexion was 1/5. Light-touch sensation was reduced in the lower extremities and below approximately the T10 level. Lower-extremity tone was documented as normal, clonus was absent, and Babinski responses were negative. The patient was unable to ambulate.

The T10 designation represents the clinical neurological level and is distinct from the vertebral levels addressed surgically. Vertebral levels of compression, decompression, and instrumentation do not directly determine the neurological level of injury. Although the rehabilitation documentation classified the injury as T10 AIS C, the original formal ISNCSCI worksheet and complete motor and sensory subscores were unavailable for independent reconstruction. In addition, great-toe extension and ankle plantarflexion grades were not available in the admission examination; therefore, a complete lower-extremity motor score could not be calculated.

Rehabilitation course

The patient was admitted to inpatient rehabilitation on February 22, 2026, eight days after the second surgical procedure. His rehabilitation program included at least three hours of combined physical and occupational therapy on treatment days, with a weekly minimum of approximately 15 hours of therapy, in addition to 24-hour specialized rehabilitation nursing care under physiatry supervision.

Physical therapy emphasized bed mobility, transfer training, seated and standing balance, trunk control, progressive standing tolerance, strengthening and motor-control activities, wheelchair mobility, and overground gait training with a front-wheeled walker. Occupational therapy emphasized functional transfers, dressing, bathing, toileting, upper-extremity conditioning, adaptive strategies, and task-specific activities-of-daily-living retraining. Rehabilitation nursing and case management addressed medical monitoring, skin protection, pain management, caregiver education, safety, and discharge planning.

Initial functional status at rehabilitation admission

At admission and during the early rehabilitation period, the patient was non-ambulatory and required moderate to maximal assistance for bed mobility and total or two-person assistance for transfers. Lower-extremity weakness, impaired motor control, poor balance, and his body habitus significantly limited mobility. He was dependent for wheelchair mobility, lower-body dressing, and toileting and required maximal assistance for bathing and upper-body dressing.

The neurological and functional findings together demonstrated substantial impairment at the beginning of the rehabilitation course. However, neurological impairment and functional dependence were considered separately throughout this report because assistance levels are influenced not only by muscle strength and sensation but also by balance, endurance, pain, fear of falling, compensatory strategies, and task-specific performance.

Early rehabilitation phase: week 1

During the first week, treatment focused on bed mobility, transfer training, seated balance, trunk control, and supported standing tolerance. The patient demonstrated progressive improvement in trunk control and required less assistance with bed mobility and transfers. Standing activities were introduced with substantial assistance.

Gait training was initially deferred because of limited lower-extremity activation, impaired motor control, and insufficient stability for safe stepping.

Intermediate rehabilitation phase: weeks 2-3

As therapy progressed, the patient demonstrated improved lower-extremity activation, motor control, supported-standing tolerance, and knee stability. These clinical changes allowed initiation of assisted overground gait training with a front-wheeled walker.

He initially ambulated approximately 50-70 feet with assistance. Transfers progressed to minimal assistance, and wheelchair mobility improved substantially. Functional gains were also observed in dressing and bathing, with assistance levels decreasing from maximal or dependent levels toward moderate and minimal assistance.

Formal muscle-strength, trunk-control, or balance-score thresholds used to determine readiness for gait training were not documented; initiation and progression were based on the treating therapists’ clinical assessment of strength, motor control, stability, standing tolerance, and safety.

Late rehabilitation phase: final week prior to discharge

During the final week, the patient continued to improve in mobility, coordination, endurance, and independence. He progressed to contact guard assistance for transfers and assisted ambulation. Gait distance increased to 170 feet using a front-wheeled walker with contact guard assistance. Wheelchair mobility improved to approximately 150 feet with standby assistance.

Activities of daily living also improved. The patient became independent with eating, required supervision for upper-body dressing, and required minimal assistance for lower-body dressing and bathing.

Fear of falling remained an important barrier during portions of the rehabilitation course. It intermittently affected confidence and willingness to participate in challenging mobility activities but improved with graded exposure, repetition, successful task completion, and therapist guidance.

Functional status at discharge

At discharge on March 20, 2026, the patient performed bed mobility with standby assistance and transfers with contact guard assistance using a front-wheeled walker. He completed assisted ambulation of 170 feet with a front-wheeled walker and contact guard assistance.

He was discharged home with home health services, family support, caregiver education, and training after a total inpatient rehabilitation stay of 26 days. Table 1 shows the functional progression during inpatient rehabilitation.

**Table 1:** Functional progression during inpatient rehabilitation. Functional progression observed during the inpatient rehabilitation course, demonstrating improvement in mobility, transfers, and ambulation from admission to discharge. Abbreviations: CGA = contact guard assist; Min A = minimal assist; Mod A = moderate assist; Max A = maximal assist; SBA = standby assist; FWW = front-wheeled walker; ft = feet

Domain	Admission	Early phase (week 1)	Intermediate phase (weeks 2-3)	Late phase/discharge
Bed Mobility	Max	Mod	Min	Standby
Transfers	Max (2-person assist)	Mod	Min	CGA
Ambulation	Non-ambulatory	Not initiated	50-70 ft with FWW (assist)	170 ft with FWW (CGA)
Wheelchair Mobility	Dependent	Max	Min	150 ft (standby assist)
Eating	Min	Supervision	Independent	Independent
Upper Body Dressing	Max	Mod	Min	Supervision
Lower Body Dressing	Dependent	Max	Mod	Min
Bathing	Max	Mod	Min	Min
Toileting	Dependent	Max	Mod	CGA

Standardized neurological and rehabilitation outcome measures, including serial ISNCSCI motor and sensory subscores, lower extremity motor scores, Trunk Control Test for Spinal Cord Injury, Spinal Cord Independence Measure III, Functional Independence Measure, Walking Index for Spinal Cord Injury II, Berg Balance Scale, 10-Meter Walk Test, and Six-Minute Walk Test, were not available and were not retrospectively inferred.

## Discussion

This case documents substantial early functional improvement after staged thoracic decompression and stabilization in a patient with motor-incomplete NTSCI. During 26 days of inpatient rehabilitation, the patient progressed from non-ambulatory status with extensive assistance across mobility and self-care tasks to assisted ambulation of 170 feet with a front-wheeled walker and contact guard assistance, with concurrent improvements in transfers, wheelchair mobility, and activities of daily living.

The preoperative neurological examination provides important context for interpreting this trajectory. Before the initial T1-T2 operation, the patient had intact upper-extremity strength but bilateral lower-extremity weakness in the approximately 4/5 range, impaired sensation below a thoracic level, hyperreflexia and other myelopathic findings, impaired gait, and preserved bowel and bladder function. Following the initial procedure, he subsequently experienced neurological deterioration characterized by progressive left-greater-than-right weakness, new foot drop, and eventual inability to ambulate. After the definitive T1-T7 decompression and stabilization, the rehabilitation-admission examination continued to demonstrate substantial asymmetric lower-extremity paresis, with strength ranging from 1/5 to 3/5 in the documented key muscle groups.

These findings demonstrate that the patient’s neurological course was dynamic and provide a more complete clinical context for the subsequent functional gains. Nevertheless, the available data do not permit precise quantification of neurological recovery. A complete formal preoperative ISNCSCI examination was unavailable, and serial segmental motor and sensory scores were not documented through discharge. The principal longitudinal outcome demonstrated in this case is therefore functional recovery, while the neurological examinations serve as important clinical context.

The documented AIS C classification is consistent with a motor-incomplete injury and therefore with preservation of descending motor pathways and greater potential for recovery than motor-complete SCI [3,4]. Tamai et al. reported frequent AIS conversion among patients with AIS C SCI, while Stenimahitis et al. identified baseline AIS grade and lower-extremity motor function as important predictors of later ambulation [4,5]. More recent prognostic evidence likewise identifies baseline neurological severity, neurological level, and motor function as important determinants of walking recovery, although much of this literature is derived from traumatic SCI populations [6].

The timing of the patient’s improvement is particularly important. Rehabilitation following the definitive T1-T7 procedure began only eight days after surgery. During this early postoperative period, surgical relief of mechanical compression, evolution or resolution of spinal cord edema and secondary injury processes, and spontaneous neurological recovery were likely major contributors to improvement [9,10]. These mechanisms cannot be individually quantified in this patient because serial imaging and complete neurological examinations were unavailable. The relatively short 26-day timeframe also does not support attributing the observed gains primarily to long-term structural neuroplasticity.

At the same time, neurological recovery alone does not fully describe the functional trajectory observed during inpatient rehabilitation. Rehabilitation likely helped translate the patient’s available neurological capacity into increasingly effective real-world function. Repetitive practice addressed bed mobility, transfers, sitting and standing balance, upright tolerance, assisted gait, wheelchair mobility, dressing, bathing, toileting, and other self-care activities. Rehabilitation nursing, caregiver education, medical management, and discharge planning provided additional support for safe functional progression. Comparative studies of traumatic and non-traumatic SCI demonstrate substantial functional gains during specialized rehabilitation and suggest that neurological severity may be more predictive of outcome than injury etiology alone [7,8].

Psychological factors are another clinically relevant component of this multifactorial recovery process. Fear of falling was documented during the rehabilitation course and intermittently limited the patient’s confidence and participation in mobility tasks. Fear of falling after SCI has been associated with reduced participation, autonomy, mobility, and physical activity [11,12]. More broadly, successful rehabilitation requires a patient to engage repeatedly with difficult and sometimes uncomfortable interventions, tolerate unsuccessful early attempts, and continue participating despite fear, pain, or uncertainty. A patient who is unwilling or unable to engage with recommended surgical and rehabilitative interventions may have less opportunity to translate available neurological capacity into functional performance. Conversely, willingness, motivation, and engagement should not be interpreted as biological mechanisms of spinal cord recovery; rather, they may influence treatment participation, practice intensity, and the extent to which neurological capacity is expressed functionally. In this case, confidence improved with graded exposure, repetition, therapist guidance, and successful completion of increasingly challenging mobility tasks.

The apparent difference between the T1-T7 vertebral surgical levels and the T10 neurological level is not inherently contradictory. The ISNCSCI neurological level is determined clinically and is not defined by the vertebral level of spinal compression, decompression, or instrumentation [3]. In this patient, the T10 AIS C classification was documented by the rehabilitation team. Because the original ISNCSCI worksheet was unavailable, this classification could not be independently reconstructed.

This report has several limitations. Although additional records provided a meaningful preoperative neurological baseline, the examination did not contain a complete segment-by-segment ISNCSCI assessment or formal preoperative AIS classification. Complete serial motor and sensory scores through discharge were also unavailable, preventing precise quantification of neurological recovery. Preoperative MRI images and final postoperative imaging following the T1-T7 procedure were unavailable for publication, limiting direct radiographic correlation between cord compression, decompression, and outcome. Standardized rehabilitation outcome measures were not documented, and detailed repetition counts for individual therapeutic activities were unavailable. Finally, this is a single-patient report without long-term post-discharge follow-up and therefore cannot establish causality or treatment effectiveness.

Despite these limitations, the case demonstrates a clinically meaningful neurofunctional trajectory. The available neurological findings establish the severity and evolution of the spinal cord dysfunction, while the serial rehabilitation data demonstrate substantial improvement in real-world mobility and self-care. The most balanced interpretation is that the patient’s recovery was multifactorial, reflecting surgical relief of mechanical compression, early postoperative and spontaneous neurological recovery, preserved motor pathways associated with motor-incomplete SCI, psychological engagement, and rehabilitation-facilitated functional adaptation.

## Conclusions

This case illustrates substantial early functional improvement following staged thoracic decompression and stabilization in a patient with motor-incomplete NTSCI. The available preoperative and postoperative neurological examinations provide important context for the patient’s evolving spinal cord dysfunction, while the serial rehabilitation data demonstrate progression from severe mobility dependence and inability to ambulate to assisted ambulation of 170 feet with a front-wheeled walker, along with meaningful gains in transfers, wheelchair mobility, and activities of daily living during a 26-day inpatient rehabilitation stay. The patient’s improvement was likely multifactorial. Surgical decompression and stabilization, early postoperative and spontaneous neurological recovery, preserved motor pathways, psychological engagement, and structured multidisciplinary rehabilitation likely contributed in overlapping ways. Because complete serial ISNCSCI examinations were unavailable, the relative contributions of neurological recovery and rehabilitation-facilitated functional adaptation cannot be quantified. This case therefore supports interpreting neurological and functional recovery as related but distinct components of the rehabilitation trajectory and is consistent with existing evidence supporting coordinated postoperative rehabilitation without establishing treatment causality or effectiveness.
